# 2,4-Bis[(prop-2-yn­yl)­oxy]benzaldehyde

**DOI:** 10.1107/S1600536812031637

**Published:** 2012-07-18

**Authors:** M. Esakkiammal, V. Selvarani, M. A. Neelakantan, V. Silambarasan, D. Velmurugan

**Affiliations:** a Chemistry Research Centre, National Engineering College, K. R. Nagar, Kovilpatti 628 503, India; b CAS in Crystallography and Biophysics, University of Madras, Guindy Campus, Chennai 25, India

## Abstract

In the title compound, C_13_H_10_O_3_, two prop-2-yn­yloxy groups are attached to the benzaldehyde ring at positions 2 and 6. The crystal packing features C—H⋯O inter­actions.

## Related literature

For the biological activity of benzaldehyde derivatives, see: Zhao *et al.* (2007 ▶). For related literature, see: Delogu *et al.* (2010 ▶); Ley & Bertram (2001 ▶). For hydrogen-bond motifs, see: Bernstein *et al.* (1995 ▶).
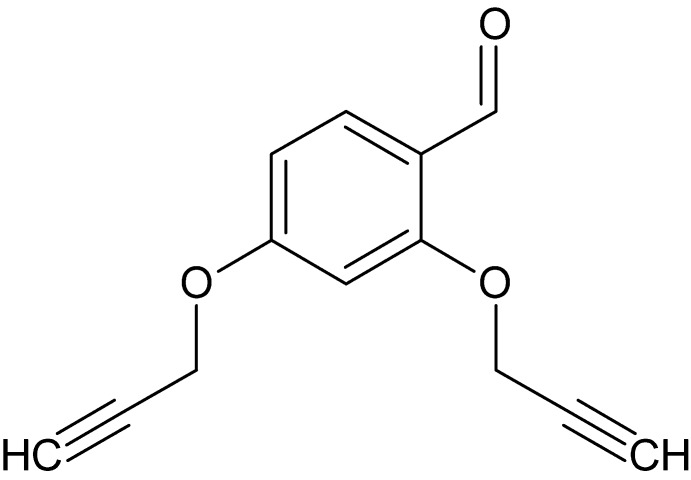


## Experimental

### 

#### Crystal data

C_13_H_10_O_3_
*M* *_r_* = 214.21
Monoclinic, 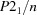

*a* = 4.9219 (2) Å
*b* = 16.8705 (7) Å
*c* = 13.4326 (6) Å
β = 98.236 (3)°
*V* = 1103.87 (8) Å^3^
*Z* = 4
Mo *K*α radiation
μ = 0.09 mm^−1^
*T* = 293 K
0.20 × 0.20 × 0.20 mm

#### Data collection

Bruker SMART APEXII area-detector diffractometer
Absorption correction: multi-scan (*SADABS*; Bruker, 2008 ▶) *T*_min_ = 0.982, *T*_max_ = 0.982
10446 measured reflections
2754 independent reflections
2177 reflections with *I* > 2σ(*I*)
*R*_int_ = 0.025

#### Refinement

*R*[*F* ^2^ > 2σ(*F* ^2^)] = 0.039
*wR*(*F* ^2^) = 0.113
*S* = 1.04
2754 reflections
154 parameters
H atoms treated by a mixture of independent and constrained refinement
Δρ_max_ = 0.25 e Å^−3^
Δρ_min_ = −0.16 e Å^−3^

### 

Data collection: *APEX2* (Bruker, 2008 ▶); cell refinement: *APEX2*; data reduction: *SAINT* (Bruker, 2008 ▶); program(s) used to solve structure: *SHELXS97* (Sheldrick, 2008 ▶); program(s) used to refine structure: *SHELXL97* (Sheldrick, 2008 ▶); molecular graphics: *ORTEP-3* (Farrugia, 1997 ▶); software used to prepare material for publication: *SHELXL97* and *PLATON* (Spek, 2009 ▶).

## Supplementary Material

Crystal structure: contains datablock(s) global, I. DOI: 10.1107/S1600536812031637/bt5961sup1.cif

Structure factors: contains datablock(s) I. DOI: 10.1107/S1600536812031637/bt5961Isup2.hkl

Supplementary material file. DOI: 10.1107/S1600536812031637/bt5961Isup3.cml

Additional supplementary materials:  crystallographic information; 3D view; checkCIF report

## Figures and Tables

**Table 1:** Hydrogen-bond geometry (Å, °)

*D*—H⋯*A*	*D*—H	H⋯*A*	*D*⋯*A*	*D*—H⋯*A*
C6—H6⋯O1^i^	0.93	2.48	3.3616 (14)	159

Symmetry code: (i) 
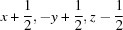
.

**Fig. 1.**
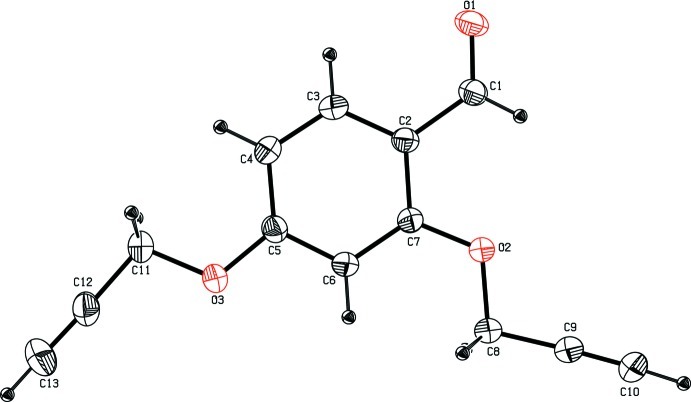
The molecular structure of the title compound, showing the atomic numbering and displacement ellipsoids drawn at 30% probability level.

**Fig. 2.**
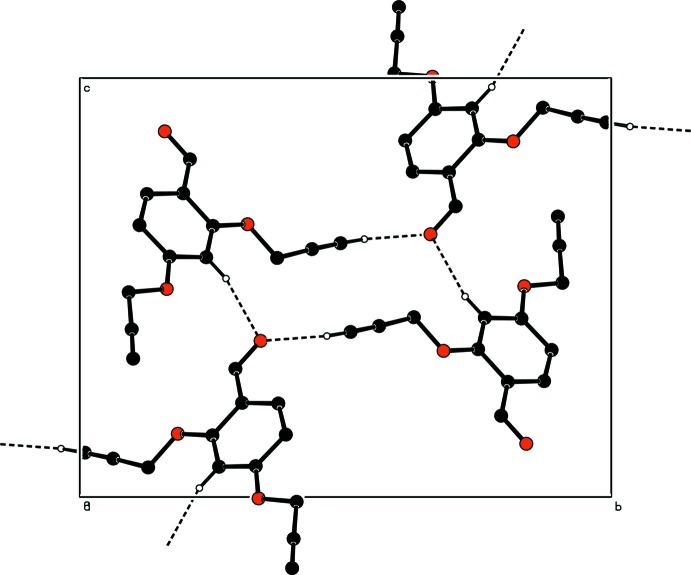
The crystal packing of the molecules viewed down a axis.

## supplementary crystallographic information

### Comment

The Schiff base derived from amines and substitued benzaldehydes exhibit antibacterial, anticancer and antitumour activities (Zhao *et al.* (2007)). Several benzaldoximes, benzaldehyde-*O*-ethyloximes, and acetophenonoximes were synthesized and evaluated as tyrosinase inhibitors (Ley & Bertram (2001)). The bis-salicylaldehydes exhibited greater inhibitory activity than salicylaldehyde (Delogu *et al.*(2010)).

The *ORTEP* plot of the molecule is shown in Fig. 1. The dihedral angles of phenyl ring (C2—C7) attached to prop-2-yn-1-yloxy group at 2, 6-positions (O2/C8/C9/C10) & (O3/C11/C12/C13) are 82.3 (1)° & 71.4 (1)°, respectively. The prop-2-yn-1-yloxy group is in an extended conformation which can be seen from torsion angles O2/C8/C9/C10= -177.0 (10)° and O3/C11/C12/C13= 166 (6)°, respectively.

The crystal packing includes an inter-molecular interaction between a terminal ethynyl H atom and an ethynyl group on a glide-related molecule and another interaction between an O-atom-linked methylene H and an ethynyl group of a different glide-related molecule.

The packing of the molecules viewed down *a* axis is shown in Fig. 2. The molecules are stabilized by C—H···π and bifurcated C—H···O types of intra and intermolecular interactions, which form a dimer C8 chain running along the *a* axis (Bernstein *et al.*, 1995).

### Experimental

2,4-dihydroxybenzaldehyde (10 mmol), 3-bromopropyne (20 mmol) and potassium carbonate (15 mmol) were suspended in acetonitrile (40 ml) and refluxed for 30 h in presence of KI (0.1 g) as catalyst. The reaction mixture was filtered while hot to remove insoluble impurities, neutralized with dil.HCl (3 N) and extracted with chloroform and dried with Na_2_SO_4_. The extracts were concentrated to obtain a brown solid which was then purified by column chromatography over SiO_2_ by eluting a mixture of 4% ethyl acetate with n-hexane. Evaporation of the purified extract yielded 2, 4-dipropynoxybenzaldehyde in the form of pure white solid. Yield: 85%. Crystals suitable for X-ray analysis were obtained by slow evaporation method.

### Refinement

H atoms were positioned geometrically (C–H = 0.93–0.97 Å) and allowed to ride on their parent atoms, with *U*_iso_(H) = 1.2*U*_eq_(C).

### Crystal data

**Table:**

C_13_H_10_O_3_	*F*(000) = 448
*M**_r_* = 214.21	*D*_x_ = 1.289 Mg m^−^^3^
Monoclinic, *P*2_1_/*n*	Mo *K*α radiation, λ = 0.71073 Å
Hall symbol: -P 2yn	Cell parameters from 2754 reflections
*a* = 4.9219 (2) Å	θ = 2.0–28.4°
*b* = 16.8705 (7) Å	µ = 0.09 mm^−^^1^
*c* = 13.4326 (6) Å	*T* = 293 K
β = 98.236 (3)°	Block, colourless
*V* = 1103.87 (8) Å^3^	0.20 × 0.20 × 0.20 mm
*Z* = 4	

### Data collection

**Table:**

Bruker SMART APEXII area-detector diffractometer	2754 independent reflections
Radiation source: fine-focus sealed tube	2177 reflections with *I* > 2σ(*I*)
Graphite monochromator	*R*_int_ = 0.025
ω and φ scans	θ_max_ = 28.4°, θ_min_ = 2.0°
Absorption correction: multi-scan (*SADABS*; Bruker, 2008)	*h* = −6→6
*T*_min_ = 0.982, *T*_max_ = 0.982	*k* = −22→22
10446 measured reflections	*l* = −16→17

### Refinement

**Table:**

Refinement on *F*^2^	Secondary atom site location: difference Fourier map
Least-squares matrix: full	Hydrogen site location: inferred from neighbouring sites
*R*[*F*^2^ > 2σ(*F*^2^)] = 0.039	H atoms treated by a mixture of independent and constrained refinement
*wR*(*F*^2^) = 0.113	*w* = 1/[σ^2^(*F*_o_^2^) + (0.0546*P*)^2^ + 0.1741*P*] where *P* = (*F*_o_^2^ + 2*F*_c_^2^)/3
*S* = 1.04	(Δ/σ)_max_ < 0.001
2754 reflections	Δρ_max_ = 0.25 e Å^−^^3^
154 parameters	Δρ_min_ = −0.16 e Å^−^^3^
0 restraints	Extinction correction: *SHELXL97* (Sheldrick, 2008), Fc^*^=kFc[1+0.001xFc^2^λ^3^/sin(2θ)]^-1/4^
Primary atom site location: structure-invariant direct methods	Extinction coefficient: 0.035 (4)

### Special details

**Table:**

Geometry. All e.s.d.'s (except the e.s.d. in the dihedral angle between two l.s. planes) are estimated using the full covariance matrix. The cell e.s.d.'s are taken into account individually in the estimation of e.s.d.'s in distances, angles and torsion angles; correlations between e.s.d.'s in cell parameters are only used when they are defined by crystal symmetry. An approximate (isotropic) treatment of cell e.s.d.'s is used for estimating e.s.d.'s involving l.s. planes.
Refinement. Refinement of *F*^2^ against ALL reflections. The weighted *R*-factor *wR* and goodness of fit *S* are based on *F*^2^, conventional *R*-factors *R* are based on *F*, with *F* set to zero for negative *F*^2^. The threshold expression of *F*^2^ > σ(*F*^2^) is used only for calculating *R*-factors(gt) *etc*. and is not relevant to the choice of reflections for refinement. *R*-factors based on *F*^2^ are statistically about twice as large as those based on *F*, and *R*- factors based on ALL data will be even larger.

### Fractional atomic coordinates and isotropic or equivalent isotropic displacement parameters (Å^2^)

**Table:**

	*x*	*y*	*z*	*U*_iso_*/*U*_eq_	
O2	0.05286 (17)	0.31557 (5)	0.65108 (6)	0.0445 (2)	
O3	0.68295 (18)	0.16361 (5)	0.49674 (7)	0.0501 (3)	
O1	0.0285 (2)	0.15987 (5)	0.87280 (7)	0.0554 (3)	
C6	0.3759 (2)	0.23786 (7)	0.57231 (9)	0.0396 (3)	
H6	0.3799	0.2751	0.5214	0.047*	
C2	0.2097 (2)	0.19456 (6)	0.72497 (8)	0.0376 (3)	
C3	0.3673 (2)	0.12642 (7)	0.72331 (9)	0.0430 (3)	
H3	0.3642	0.0890	0.7739	0.052*	
C4	0.5284 (2)	0.11234 (7)	0.64902 (9)	0.0447 (3)	
H4	0.6320	0.0662	0.6492	0.054*	
C9	−0.1318 (3)	0.43691 (7)	0.59180 (9)	0.0447 (3)	
C7	0.2148 (2)	0.25048 (6)	0.64716 (8)	0.0361 (2)	
C5	0.5317 (2)	0.16901 (7)	0.57387 (9)	0.0398 (3)	
C12	0.9705 (3)	0.09766 (8)	0.40030 (11)	0.0554 (3)	
C1	0.0455 (3)	0.20717 (7)	0.80598 (9)	0.0459 (3)	
H1	−0.0521	0.2544	0.8063	0.055*	
C10	−0.2730 (3)	0.49054 (8)	0.60585 (11)	0.0539 (3)	
C11	0.8314 (3)	0.09202 (8)	0.48871 (10)	0.0495 (3)	
H11A	0.7066	0.0472	0.4822	0.059*	
H11B	0.9646	0.0844	0.5485	0.059*	
C8	0.0455 (3)	0.37140 (7)	0.57073 (9)	0.0453 (3)	
H8A	−0.0264	0.3465	0.5073	0.054*	
H8B	0.2290	0.3907	0.5663	0.054*	
C13	1.0861 (4)	0.10046 (11)	0.33041 (14)	0.0791 (5)	
H10	−0.379 (4)	0.5352 (11)	0.6156 (14)	0.087 (6)*	
H13	1.176 (5)	0.1042 (13)	0.2780 (18)	0.114 (7)*	

### Atomic displacement parameters (Å^2^)

**Table:**

	*U*^11^	*U*^22^	*U*^33^	*U*^12^	*U*^13^	*U*^23^
O2	0.0548 (5)	0.0381 (4)	0.0443 (5)	0.0099 (4)	0.0197 (4)	0.0088 (3)
O3	0.0517 (5)	0.0511 (5)	0.0520 (5)	0.0140 (4)	0.0224 (4)	0.0074 (4)
O1	0.0824 (7)	0.0460 (5)	0.0421 (5)	−0.0069 (4)	0.0231 (5)	0.0041 (4)
C6	0.0420 (6)	0.0392 (6)	0.0391 (6)	0.0024 (5)	0.0107 (5)	0.0070 (5)
C2	0.0419 (6)	0.0363 (6)	0.0351 (5)	−0.0024 (4)	0.0070 (4)	0.0019 (4)
C3	0.0493 (7)	0.0398 (6)	0.0400 (6)	0.0020 (5)	0.0068 (5)	0.0085 (5)
C4	0.0453 (6)	0.0406 (6)	0.0484 (7)	0.0091 (5)	0.0081 (5)	0.0052 (5)
C9	0.0512 (7)	0.0403 (6)	0.0432 (6)	0.0027 (5)	0.0090 (5)	0.0061 (5)
C7	0.0376 (5)	0.0335 (5)	0.0377 (6)	0.0004 (4)	0.0072 (4)	0.0020 (4)
C5	0.0361 (5)	0.0439 (6)	0.0402 (6)	0.0021 (4)	0.0089 (4)	0.0009 (5)
C12	0.0588 (8)	0.0540 (8)	0.0555 (8)	0.0105 (6)	0.0152 (6)	−0.0073 (6)
C1	0.0597 (7)	0.0398 (6)	0.0409 (6)	−0.0003 (5)	0.0158 (5)	0.0017 (5)
C10	0.0645 (8)	0.0434 (7)	0.0551 (8)	0.0106 (6)	0.0129 (6)	0.0036 (6)
C11	0.0487 (7)	0.0486 (7)	0.0533 (7)	0.0094 (5)	0.0146 (6)	−0.0023 (6)
C8	0.0520 (7)	0.0425 (6)	0.0437 (6)	0.0085 (5)	0.0147 (5)	0.0105 (5)
C13	0.0998 (13)	0.0800 (12)	0.0659 (10)	0.0122 (10)	0.0402 (10)	−0.0076 (9)

### Geometric parameters (Å, º)

**Table:**

O2—C7	1.3622 (13)	C4—C5	1.3922 (16)
O2—C8	1.4292 (14)	C4—H4	0.9300
O3—C5	1.3626 (13)	C9—C10	1.1722 (18)
O3—C11	1.4235 (14)	C9—C8	1.4608 (16)
O1—C1	1.2127 (14)	C12—C13	1.166 (2)
C6—C7	1.3832 (15)	C12—C11	1.4565 (18)
C6—C5	1.3905 (15)	C1—H1	0.9300
C6—H6	0.9300	C10—H10	0.935 (19)
C2—C3	1.3886 (16)	C11—H11A	0.9700
C2—C7	1.4109 (15)	C11—H11B	0.9700
C2—C1	1.4611 (15)	C8—H8A	0.9700
C3—C4	1.3814 (17)	C8—H8B	0.9700
C3—H3	0.9300	C13—H13	0.89 (2)
			
C7—O2—C8	116.99 (8)	C6—C5—C4	121.39 (10)
C5—O3—C11	117.16 (9)	C13—C12—C11	178.21 (17)
C7—C6—C5	119.35 (10)	O1—C1—C2	124.02 (11)
C7—C6—H6	120.3	O1—C1—H1	118.0
C5—C6—H6	120.3	C2—C1—H1	118.0
C3—C2—C7	118.20 (10)	C9—C10—H10	176.8 (12)
C3—C2—C1	120.16 (10)	O3—C11—C12	108.22 (11)
C7—C2—C1	121.65 (10)	O3—C11—H11A	110.1
C4—C3—C2	122.26 (11)	C12—C11—H11A	110.1
C4—C3—H3	118.9	O3—C11—H11B	110.1
C2—C3—H3	118.9	C12—C11—H11B	110.1
C3—C4—C5	118.25 (11)	H11A—C11—H11B	108.4
C3—C4—H4	120.9	O2—C8—C9	107.67 (9)
C5—C4—H4	120.9	O2—C8—H8A	110.2
C10—C9—C8	177.88 (13)	C9—C8—H8A	110.2
O2—C7—C6	123.48 (9)	O2—C8—H8B	110.2
O2—C7—C2	115.97 (9)	C9—C8—H8B	110.2
C6—C7—C2	120.55 (10)	H8A—C8—H8B	108.5
O3—C5—C6	113.87 (10)	C12—C13—H13	178.1 (16)
O3—C5—C4	124.74 (10)		
			
C7—C2—C3—C4	0.52 (18)	C11—O3—C5—C4	4.98 (17)
C1—C2—C3—C4	−179.18 (11)	C7—C6—C5—O3	−179.57 (10)
C2—C3—C4—C5	0.20 (19)	C7—C6—C5—C4	0.22 (18)
C8—O2—C7—C6	2.28 (16)	C3—C4—C5—O3	179.19 (11)
C8—O2—C7—C2	−177.39 (10)	C3—C4—C5—C6	−0.58 (18)
C5—C6—C7—O2	−179.13 (10)	C3—C2—C1—O1	−2.48 (19)
C5—C6—C7—C2	0.53 (17)	C7—C2—C1—O1	177.83 (12)
C3—C2—C7—O2	178.80 (10)	C5—O3—C11—C12	178.25 (10)
C1—C2—C7—O2	−1.51 (16)	C13—C12—C11—O3	166 (6)
C3—C2—C7—C6	−0.89 (16)	C7—O2—C8—C9	−178.20 (10)
C1—C2—C7—C6	178.81 (11)	C10—C9—C8—O2	−177 (100)
C11—O3—C5—C6	−175.24 (11)		

### Hydrogen-bond geometry (Å, º)

**Table:**

*D*—H···*A*	*D*—H	H···*A*	*D*···*A*	*D*—H···*A*
C6—H6···O1^i^	0.93	2.48	3.3616 (14)	159

Symmetry code: (i) *x*+1/2, −*y*+1/2, *z*−1/2.
